# Supplementary material for: Chemical language modeling with structured state space sequence models
Source: Nat Commun. 2024 Jul 22;15:6176. doi: 10.1038/s41467-024-50469-9 (PMC11263548; doi:10.1038/s41467-024-50469-9)
Supplement: Supplementary file 1 — Supplementary Information [file 41467_2024_50469_MOESM1_ESM.pdf]

Supporting Information

Chemical Language Modeling with Structured State Space Sequence Models

Rıza Özçelik, Sarah de Ruiter, Emanuele Criscuolo, Francesca Grisoni

Dual formulation of discrete state-space models

Discrete state space models (SSMs) are typically defined with the following equations:

$$\begin{aligned} x_k &= \overline{\boldsymbol{A}} x_{k-1} + \overline{\boldsymbol{B}} u_k \\ y_k &= \overline{\boldsymbol{C}} x_k \quad + \overline{\boldsymbol{D}} u_k, \end{aligned} \tag{1}$$

where u_k is the kth element of the input sequence, x_k is the state vector after processing the input u_k, and y_k is the kth element of the output sequence. The matrices, $\overline{\boldsymbol{A}} \in \mathbb{R}^{N \times N}$, $\overline{\boldsymbol{B}} \in \mathbb{R}^{N \times 1}$, $\overline{\boldsymbol{C}} \in \mathbb{R}^{1 \times N}$, and $\overline{\boldsymbol{D}} \in \mathbb{R}^{1 \times 1}$ are the (learnable) parameters of the model. Overall, a discrete SSM defines a sequence-to-sequence mapping, where the mapping is defined via the matrices.

So, SSMs are recurrence relations and can be used for auto-regressive tasks (*e.g.,* generation), where x_k is used as the state of the recurrent computation. Figure 1c illustrates the computation diagram of the SSM defined by Equation (1), which resembles the computation graph of a recurrent neural network with a skip connection.

Setting the initial state (x_0) to 0 and writing out the Equation (1) yields the following formula for the state variable x_k:

$$\begin{aligned} x_1 &= \overline{\boldsymbol{A}} x_0 \quad + \overline{\boldsymbol{B}} u_1 = \overline{\boldsymbol{B}} u_1 \\ x_2 &= \overline{\boldsymbol{A}} x_1 \quad + \overline{\boldsymbol{B}} u_2 = \overline{\boldsymbol{A}\boldsymbol{B}} u_1 + \overline{\boldsymbol{B}} u_2 \\ x_3 &= \overline{\boldsymbol{A}} x_2 \quad + \overline{\boldsymbol{B}} u_3 = \overline{\boldsymbol{A}}^2 \overline{\boldsymbol{B}} u_1 + \overline{\boldsymbol{A}\boldsymbol{B}} u_2 + \overline{\boldsymbol{B}} u_3 \\ &\vdots \\ x_k &= \overline{\boldsymbol{A}} x_{k-1} + \overline{\boldsymbol{B}} u_k = \overline{\boldsymbol{A}}^{k-1} \overline{\boldsymbol{B}} u_1 + \overline{\boldsymbol{A}}^{k-2} \overline{\boldsymbol{B}} u_2 + \cdots + \overline{\boldsymbol{B}} u_k. \end{aligned} \tag{2}$$

Setting $\overline{\boldsymbol{D}} = 0$ (*i.e.,* disabling the skip connection from input to output), the output variable $y_k = \overline{\boldsymbol{C}} x_k + \overline{\boldsymbol{D}} u_k$ becomes:

$$y_k = \overline{\boldsymbol{C}\boldsymbol{A}}^{k-1} \overline{\boldsymbol{B}} u_1 + \overline{\boldsymbol{C}\boldsymbol{A}}^{k-2} \overline{\boldsymbol{B}} u_2 + \cdots + \overline{\boldsymbol{C}\boldsymbol{B}} u_k. \tag{3}$$

Defining $\overline{\boldsymbol{K}}^i = \overline{\boldsymbol{C}\boldsymbol{A}}^i \overline{\boldsymbol{B}}$, Equation (3) becomes:

$$y_k = \sum_i \overline{\boldsymbol{K}}^{\boldsymbol{i}} u_{k-i} = u * \overline{\boldsymbol{K}}, \tag{4}$$

where $*$ is the convolution operator. The derivation shows that the parameters that define the recurrence relation of an SSM can also define a convolution with an unbounded window. Therefore, an SSM can be formulated as a global convolution during training time to capture long-range dependencies and benefit from accelerated training in GPUs. During the test time, the learned matrices can be used to define a recurrence relation for fast auto-regressive generation.

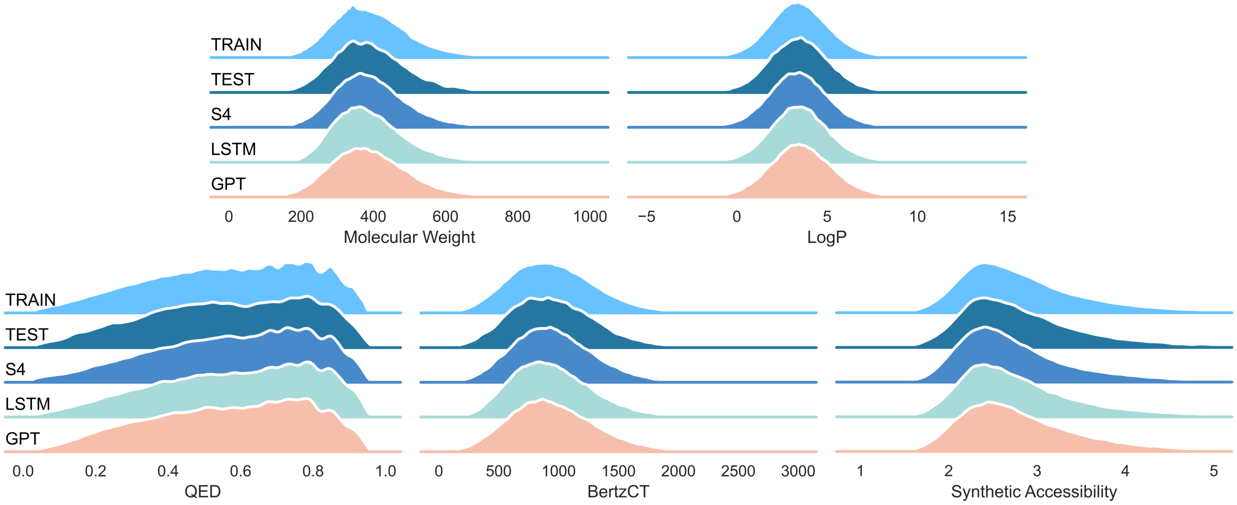

Supplementary Fig. 1: *Molecular descriptor distribution of designs after pre-training.* All models are sampled 102,400 designs and molecular weight, octanol-water partition coefficient (Log P), quantitative estimate of drug-likeness (QED), Bertz complexity (BertzCT), and synthetic accessibility are computed. Source data are provided as a Source Data file.

Supplementary Table 1: *Summary statistics of designs after pre-training.* All models are sampled 102,400 designs and molecular weight, octanol-water partition coefficient (Log P), quantitative estimate of drug-likeness (QED), Bertz complexity (BertzCT), and synthetic accessibility are computed. The mean and standard deviation of the descriptors are reported.

**Descriptor**	**S4**	**LSTM**	**GPT**	**Training**	**Test**
Molecular Weight	395 ± 105	392 ± 100	392 ± 109	395 ± 109	395 ± 109
LogP	3.40 ± 1.83	3.49 ± 1.77	3.59 ± 1.84	3.46 ± 1.80	3.47 ± 1.80
QED	0.58 ± 0.21	0.58 ± 0.21	0.57 ± 0.21	0.57 ± 0.22	0.56 ± 0.22
BertzCT	956 ± 339	948 ± 336	954 ± 363	956 ± 361	957 ± 363
Synthetic Accessibility	2.81 ± 0.75	2.86 ± 0.78	2.85 ± 0.74	2.89 ± 0.79	2.89 ± 0.80

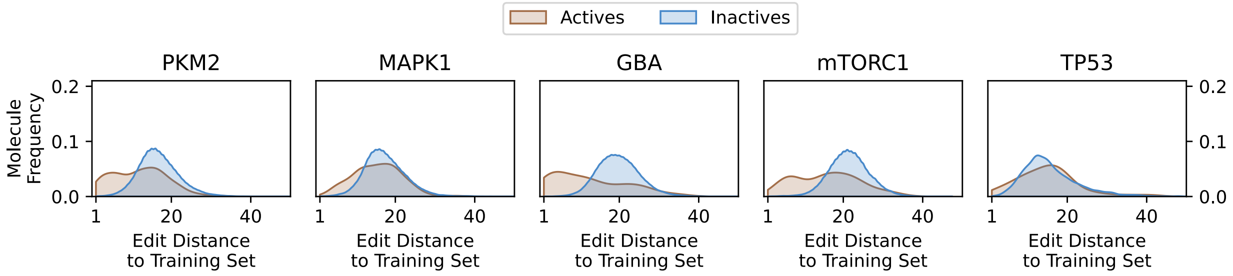

Supplementary Fig. 2: *Similarity of test set molecules to the training set.* Test sets across ten data splits are pooled together and the minimum edit distance of each test set molecule to the respective training set is computed per actives and ianctives. Source data are provided as a Source Data file.

Supplementary Table 2: *Benchmarking S4 using MOSES [51].* A total of 107 models were trained with different hyper-parameters, and used to sample 102,400 SMILES strings. Here, the statistics of the model with the best trade-off between FCD/TestSF and novelty trade-off was reported for comparison. The best three deep learning approaches per metric are highlighted in boldface. S4 is systematically in the top-3 deep learning approaches across all metrics, except for validity (where it is in the top-5).

Model	Valid (%)	Unique@1k (%)	Unique@10k (%)	Novelty (%)	IntDiv (↑)	FCD (↓)	
						Test	TestSF
Train	*n.a.*	*n.a.*	*n.a.*	*n.a.*	0.86	0.01	0.48
HMM	7.6	62.3	56.7	99.9	0.85	24.5	25.4
NGram	23.8	97.4	92.2	96.9	0.87	5.51	6.23
Combinatorial	100.0	99.8	99.1	98.8	0.87	4.24	4.51
CharRNN	97.5	**100.0**	99.9	84.2	**0.86**	**0.07**	**0.52**
AAE	93.7	**100.0**	99.7	79.3	**0.86**	0.56	1.06
VAE	97.7	**100.0**	99.8	69.5	**0.86**	**0.10**	0.57
JTN-VAE	**100.0**	**100.0**	**100.0**	**91.4**	0.85	0.40	0.94
LatentGAN	89.7	**100.0**	**100.0**	**95.0**	**0.86**	0.30	0.83
MD-TF [9]	**99.6**	100.0	99.9	81.6	0.85	0.11	**0.51**
cMolGPT [10]	98.8	100.0	99.9	-	-	-	-
TD-GPT [11]	**99.3**	100.0	99.4	78.1	-	-	-
S4 (this paper)	98.4	**100.0**	**100.0**	**88.1**	**0.86**	**0.08**	**0.43**

Supplementary Table 3: *Number of compounds used during the transfer-learnig phase.* Bioactive molecules were extracted from LIT-PCBA [53] database per each target and randomly divided into a training (80%), validation (10%) and test set (10%). Additionally, the test set contained 10,240 inactive molecules per target (chosen by random sampling). For TP53, all the inactive molecules available in the original dataset were considered.

**Dataset**	**Train**	**Valid.**	**Test**	
			Active	*Inact.*
PKM2	436	54	56	10,240
MAPK1	246	30	32	10,240
GBA	132	16	18	10,240
mTORC1	77	9	11	10,240
TP53	44	10	10	3,301

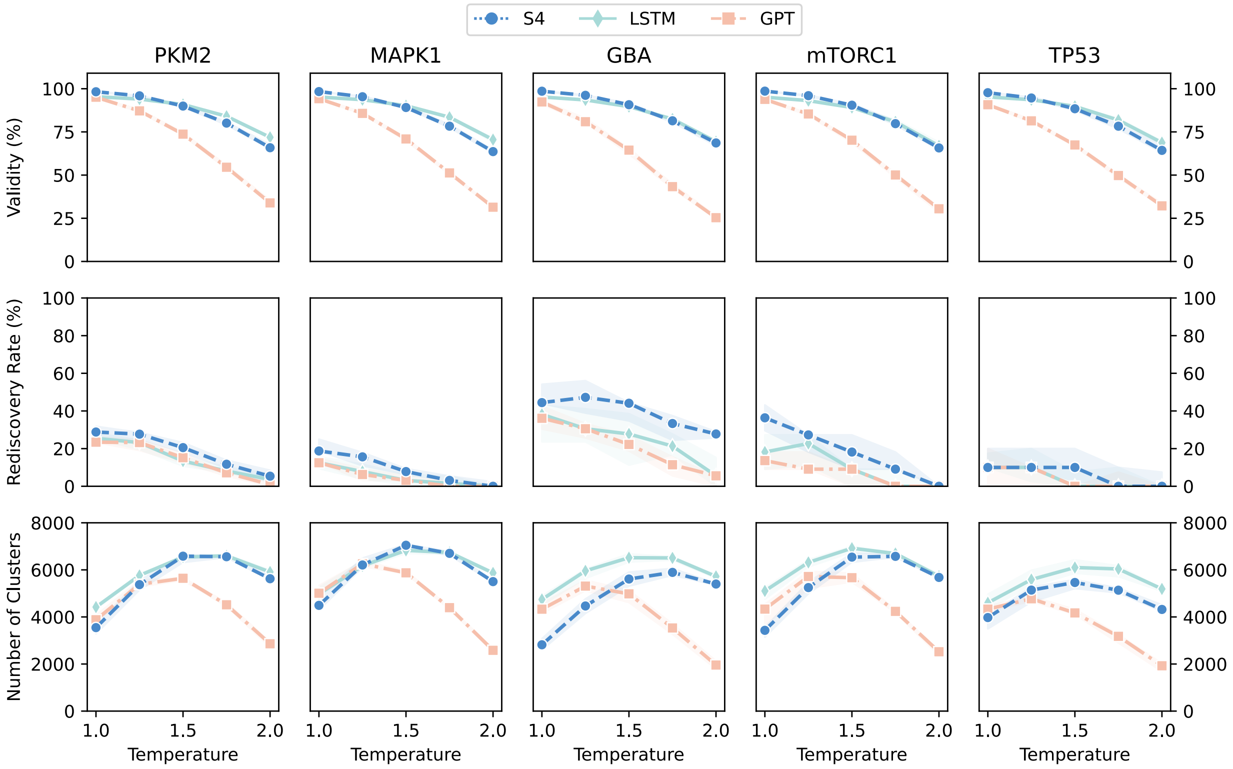

Supplementary Fig. 3: *Chemical space exploration per protein target.* The models were sampled in temperatures between 1 and 2 and validity, rediscovery rate (similarity above 60%), and number of scaffold clusters were computed. Source data are provided as a Source Data file.

Supplementary Table 4: *Hyper-parameter candidates for the architectures.* The combinatorial space of hyper-parameters is defined via the values in the table. A random search strategy is used to sample the space and conduct the experiments.

Hyper-parameter	LSTM	GPT	S4 - Stage 1	S4 - Stage 2
LSTM layers	(64, 64, 64), (64, 64), (128, 64, 32), (128, 128), (128, 128, 128), (256, 128), (256, 128, 64), (256, 256), (1024, 512), (2048)	NA	NA	NA
# transformer blocks	NA	1, 2, 4, 6	NA	NA
# S4 blocks	NA	NA	1, 2, 4, 6, 8	4, 8
Model dimension	NA	NA	64, 128, 256	256, 512
Number of SSMs	NA	NA	1	1, 2, 4
Feed-forward dimension	NA	64, 128, 256	NA	NA
Embedding size	64, 128, 256	32, 64, 128, 256	64, 128, 256	256, 512
Dropout rate	0.0, 0.1, 0.2	0.0, 0.1, 0.2	0.0, 0.1, 0.2	0.0, 0.25
Learning rate	1e-2, 1e-3, 5e-4	1e-2, 1e-3, 5e-4	1e-2, 1e-3, 5e-4	1e-2, 1e-3, 5e-3
Batch size	64, 128, 512, 1024	64, 128, 512, 1024	64, 128, 512, 1024	2048
Embedding dropout	NA	NA	0.0, 0.1, 0.2	0.25
Softmax dropout	NA	NA	0.0, 0.1, 0.2	0.0

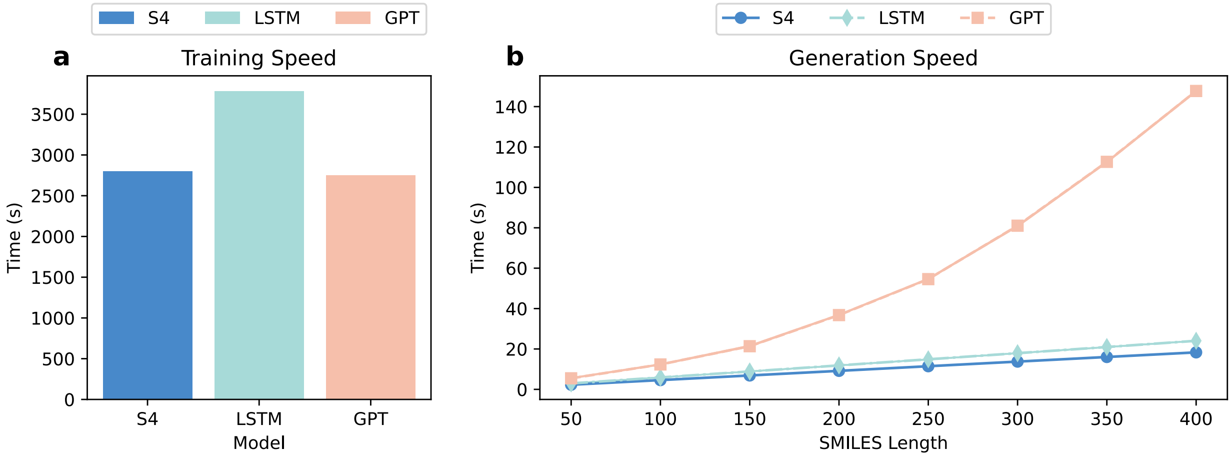

Supplementary Fig. 4: *Computational efficiency of training and molecule generation.* (a) 10 models are trained on sequences of length 450 and the mean training time is reported. (b) 10,240 designs are generated with 10 repetitions and mean generation time is measured in increasing lengths. A separate GPT model is trained per experimented SMILES length. Compute times are computed using an NVIDIA A100 40GB cloud GPU for both plots, and standard deviations are omitted for being negligible. Source data are provided as a Source Data file.
